# Capsomer Vaccines Protect Mice from Vaginal Challenge with Human Papillomavirus

**DOI:** 10.1371/journal.pone.0027141

**Published:** 2011-11-01

**Authors:** Wai-Hong Wu, Elizabeth Gersch, Kihyuck Kwak, Subhashini Jagu, Balasubramanyam Karanam, Warner K. Huh, Robert L. Garcea, Richard B. S. Roden

**Affiliations:** 1 Department of Pathology, Johns Hopkins University, Baltimore, Maryland, United States of America; 2 Department of Molecular, Cellular and Developmental Biology, University of Colorado, Boulder, Colorado, United States of America; 3 Department of Gynecologic Oncology, University of Alabama at Birmingham, Birmingham, Alabama, United States of America; 4 Department of Oncology, Johns Hopkins University, Baltimore, Maryland, United States of America; 5 Department of Gynecology and Obstetrics, Johns Hopkins University, Baltimore, Maryland, United States of America; National Institute of Health - National Cancer Institute, United States of America

## Abstract

Capsomers were produced in bacteria as glutathione-S-transferase (GST) fusion proteins with human papillomavirus type 16 L1 lacking the first nine and final 29 residues (GST-HPV16L1Δ) alone or linked with residues 13–47 of HPV18, HPV31 and HPV45 L2 in tandem (GST-HPV16L1Δ-L2x3). Subcutaneous immunization of mice with GST-HPV16L1Δ or GST-HPV16L1Δ-L2x3 in alum and monophosphoryl lipid A induced similarly high titers of HPV16 neutralizing antibodies. GST-HPV16L1Δ-L2x3 also elicited moderate L2-specific antibody titers. Intravaginal challenge studies showed that immunization of mice with GST-HPV16 L1Δ or GST-HPV16L1Δ-L2x3 capsomers, like Cervarix®, provided complete protection against HPV16. Conversely, vaccination with GST-HPV16 L1Δ capsomers failed to protect against HPV18 challenge, whereas mice immunized with either GST-HPV16L1Δ-L2x3 capsomers or Cervarix® were each completely protected. Thus, while the L2-specific response was moderate, it did not interfere with immunity to L1 in the context of GST-HPV16L1Δ-L2x3 and is sufficient to mediate L2-dependent protection against an experimental vaginal challenge with HPV18.

## Introduction

Persistent infection by high risk HPV types is a necessary, but not sufficient cause of cervical cancer, as well as a significant fraction of other anogenital cancers and subset of head and neck cancers [1]. Two licensed HPV vaccines, Gardasil® (Merck) and Cervarix® (GSK), are derived from the viral major capsid protein L1, which assembles into virus-like particles (VLPs) when expressed in eukaryotic cells [2]. Gardasil® is derived from L1 expression in yeast and Cervarix® from expression in baculovirus-infected insect cells. In Gardasil®, L1 VLPs derived from HPV6 (20 µg), HPV11 (40 µg), HPV16 (40 µg) and HPV18 (20 µg) are formulated using an amorphous aluminum hydroxyphosphate sulphate adjuvant (225 µg). In Cervarix®, HPV16 and HPV18 L1 VLPs (20 µg each) are adjuvanted with aluminum hydroxide (500 µg) and 3-O-desacyl-4′-monophosphoryl lipid A (MPL, 50 µg). Both vaccines have demonstrated remarkable protective efficacy against infection by the specific HPV types present in each vaccine [3], [4], [5], [6]. Importantly, efficacy has been demonstrated against the development of low- and high-grade intraepithelial neoplasia at the cervix, vaginal, vulva and anus that are associated with HPV16 and HPV18 infection [7]. Vaccination with HPV6 and HPV11 VLPs also prevents the occurrence of benign genital warts caused by these types [8]. Vaccination with L1 VLPs induces type-restricted immunity, *i.e.,* near absolute protection against the type utilized to generate the vaccine, significant but weaker cross-protection for the most closely phylogenetically-related types (*e.g.,* HPV16 and HPV31, or HPV18 and HPV45), and partial to no protection against more distantly related types or types from other species [6], [9]. HPV16 and HPV18 are associated with 50% and 20% of cervical cancer cases, respectively. Because more than a dozen high risk types are associated with cervical cancer and benign genital warts cause significant morbidity, there is an effort to develop a nonavalent L1 VLP vaccine targeting the seven most common oncogenic HPV types and the two most common types in genital warts, HPV6 and HPV11 [10], [11].

High-risk HPV infection causes ≈5% of all cancer worldwide, but ≈85% of cervical cancer cases occur in low income countries, reflecting their lack of resources and infrastructure to support national cytologic screening efforts [12]. Therefore, the potential benefits of HPV vaccination are likely to be greatest in low resource settings, highlighting the need to develop inexpensive and broadly protective HPV vaccines that can be delivered globally [2].

VLPs are formed by the assembly of seventy-two capsomers, and each capsomer is comprised of five L1 molecules [13], [14]. L1 capsomers are potential low cost alternatives to VLPs because they can be purified after expression in bacteria in high yields [15], [16], [17]. Deletion of the N-terminal 9 amino acids and the C-terminal 29 amino acids from L1 (herein abbreviated as L1Δ) enhances the yield of capsomers [16], [18].

Vaccination of dogs with GST-fused canine oral papillomavirus (COPV) L1 capsomers protects against experimental oral challenge with COPV [19]. Vaccination with COPV L1 is completely protective without an adjuvant using only 400 ng capsomers or 50 ng VLPs. Passive transfer of serum immunoglobulins from COPV L1 VLP-vaccinated dogs to naïve recipients protected the latter from experimental COPV challenge, indicating the capacity of neutralizing antibody to mediate protection. Capsomers display comparable type-restricted neutralizing epitopes to L1 VLPs [20], [21] , but low avidity and broadly reactive L1 epitopes have been described [22]. Because they are not coordinated with other capsomers and are deleted at both termini, we hypothesized that L1Δ capsomers potentially may display cross-protective epitopes that are otherwise hidden in VLPs and trigger low avidity broadly protective antibodies difficult to detect by neutralization assays [23]. Herein we examine the potential of L1Δ capsomers to generate antibodies that are both broadly neutralizing and protective against vaginal challenge.

The need for broad protection against high-risk HPV types can potentially be provided by multivalent combinations of capsomers, but this formulation complicates manufacture. As an alternative, we explored the C-terminal fusion of the minor capsid protein L2 to L1Δ, a site on the capsomer that tolerates such additions without adversely affecting its structure. Residues at the amino terminus of L2, including amino acids 13–47 [24], [25], [26], contain broadly protective epitopes [24], [27], [28], but they are weakly immunogenic in comparison to L1 [29], [30]. Indeed, when L2 is co-assembled with L1 in a VLP, L2 is unrecognized by the immune system either because of its internal positioning or the context of L1 dominant epitopes [29]. We hypothesized that the tandem fusion of L2 residues 13–47 from three different and common high-risk HPV types (derived from HPV18, HPV31 and HPV45 that cause 17.2%, 2.9% and 6.7% of cervical cancer cases respectively [10]) to the C-terminus of HPV16 L1Δ (the type responsible for 53.9% of cervical cancer cases) would enhance the immunogenicity of L2 by displaying a dense and regular array of epitopes [31], and thus provide broad protection (potentially >80%) against cervical cancer using a single antigen.

## Results

### Capsomer construction and vaccination

The generation of the HPV16 L1 deletion mutant (GST-HPV16L1Δ) and its purification after expression as a GST fusion protein in bacteria have been previously described [16]. A second construct (GST-HPV16L1Δ-L2x3) was prepared for this study by fusing a bacterial codon-optimized sequence encoding L2 amino acids 13–47 of HPV18 with the corresponding sequences of HPV31 and HPV45 in tandem to the C-terminus of GST-HPV16L1Δ. Both constructs were used for the expression of high levels of soluble protein in bacteria. These proteins were purified from lysates by affinity FPLC chromatography with glutathione sepharose. A ∼5-fold lower dose of L1 as VLP compared to capsomers is sufficient to induce immunity [19], [32].Therefore, to assess the immunogenicity of the GST-HPV16L1Δ and GST-HPV16L1Δ-L2x3 capsomers, groups of Balb/c mice were immunized three times at two-week intervals with 25 µg of capsomers formulated in 50 µg alum and 5 µg MPL per dose. For comparison, an additional group of mice was vaccinated with 1/10^th^ of a human dose of Cervarix®, which was comprised of 2 µg each of HPV16 and HPV18 L1 VLPs formulated in 50 µg alum and 5 µg MPL.

### Serum neutralization antibody titers

**Figure 1:**
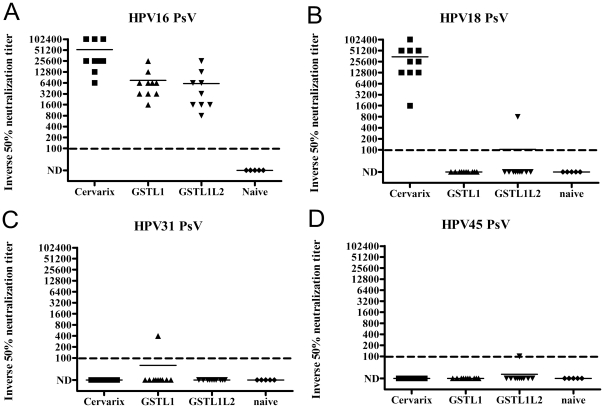
**Neutralizing antibody responses of mice vaccinated with capsomers.** Two weeks after the third immunization with GST-HPV16L1Δ, GST-HPV16L1Δ-L2x3 or Cervarix®, two-fold dilutions of serum from naïve or immunized mice were tested for *in vitro* neutralization activity against pseudovirions derived from HPV16 (A), HPV18 (B), HPV31 (C) or HPV45 (D) starting at a dilution of 1∶100. The reciprocal of the highest serum dilution with ≥50% reduction in reporter gene expression signal was defined as the serum virus neutralizing antibody titer, and <100 was considered not detected (ND) and arbitrarily plotted at 10.

Two weeks after the third immunization, serum samples from each immunized group were tested for neutralizing antibody titers against the four HPV types most commonly found in cervical cancer [33]. HPV16-specific serum neutralization titers were detected in all groups of mice except for the naïve group (Figure 1). Animals immunized with Cervarix® showed a very high mean serum titer of HPV16 neutralizing antibodies (53,120), whereas the GST-HPV16-L1Δ and GST-HPV16-L1Δ-L2x3 capsomer-immunized groups exhibited mean titers of 7520 and 6160, respectively (Figure 1A), suggesting that the greater immunogenicity of VLP may provide dose sparing as compared with capsomers. However, the average anti-HPV18, HPV31 and HPV45 neutralization titers were below the limit of detection (<100) for both groups that received capsomer vaccines, which is consistent with type-restricted neutralization (Figures 1B-D). Serum samples from the Cervarix®-immunized group exhibited an average neutralization titer of 34,720 against HPV18, whereas average neutralization titers against HPV31 and HPV45 were below the limit of detection (<100). The latter finding is surprising because vaccination with Cervarix® protects patients against HPV31 and HPV45 [6] and modest titers of neutralizing antibodies for these types were detected in some vaccinated patients [34], indicating that even very low titers of neutralizing antibodies are protective.

### Serum anti-HPV L2 ELISA titers

**Figure 2:**
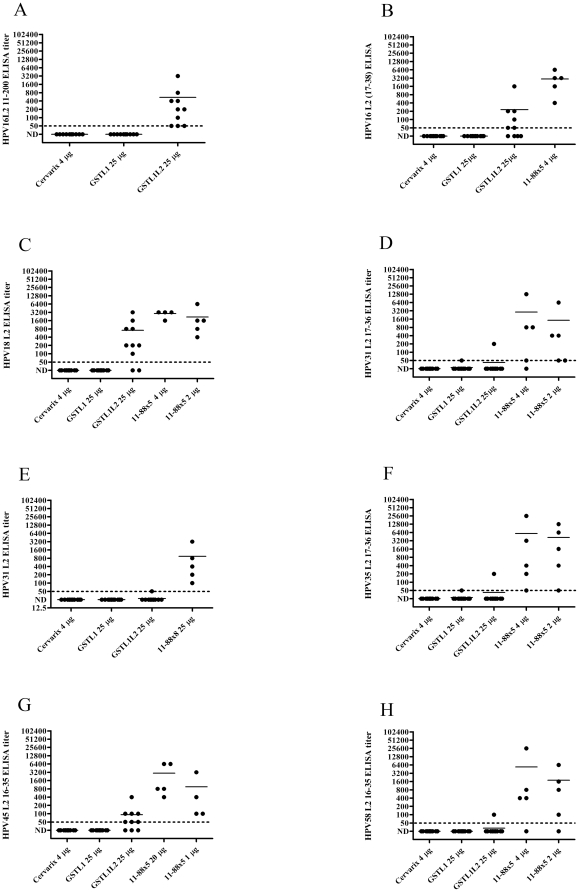
**L2-specific antibody titers induced by vaccination of mice with capsomers.** Two weeks after the third immunization with GST-HPV16L1Δ, GST-HPV16L1Δ-L2x3 or Cervarix®, two-fold dilutions of serum from immunized mice were tested by ELISA for L2 specific antibodies. Microtiter plates were coated with either HPV16 L2 amino acids 11–200 (A), or the 17–38 L2 peptide (B), or HPV18 L2 full length protein (C), or HPV31 L2 17–36 peptide (D), or HPV31 full length protein (E) or HPV35 L2 17–36 peptide (F), or HPV45 L2 16–35 peptide (G), or HPV58 L2 16–35 peptide (H), and then incubated with diluted serum samples (1∶50 as the initial dilution; two-fold serial dilutions).

Serum samples from each immunized group taken two weeks after the third immunization were tested for L2-specific antibody titers by ELISA against either HPV16 L2 11–200 or HPV16 L2 17–36, conserved neutralizing and protective epitopes [24], [35]. Sera of mice vaccinated with GST-HPV16L1Δ-L2x3 exhibited mean ELISA titers of 545 and 230, respectively, against HPV16 L2 11–200 and 17–38 (Figure 2). Since the GST-HPV16L1Δ-L2x3 contains L2 sequences derived from HPV18, 31 and 45, we examined the reactivity of the sera to full length HPV18 L2 protein, HPV31 full length L2 protein and 17–36 peptide, and HPV45 L2 16–35 peptide. The GST-HPV16L1Δ-L2x3 antisera reacted specifically with the HPV18 and HPV45 L2 polypeptides, but not with either the HPV31 full length protein or the 17–36 peptide. The GST-HPV16L1Δ-L2x3 antisera also failed to react with HPV35 L2 17–36 or HPV58 L2 16–35 peptides, two types that were not included in the vaccine construct.

We previously showed that the L1 and L2-specific antibody responses to vaccination with L1 capsomers mixed with multimeric L2 protein 11-88x5 (a fusion of L2 amino acid residues 11–88 derived from HPV1, 5, 6, 16 and 18) were independent (*i.e.,* no interference was noted) [21]. Although fusion of L2 with L1 capsomers did not significantly impact the neutralizing antibody response, surprisingly, no neutralizing antibody titers were detected for HPV18, HPV31 or HPV45, indicating that the L1-specific response might dominate the L2-specific response to GST-16L1Δ-L2x3 vaccination. GST-HPV16L1Δ-L2x3 is a fusion peptide that contains GST, an L1 deletion mutant and three copies of L2 amino acids 13 to 47 derived from HPV types 18, 31 and 45, while 11-88x5 is a fusion of L2 amino acids 11 to 88 from five different HPV types (HPV1, 5, 6, 16 and 18) [36]. Thus, vaccination with 4 µg of the latter would provide a similar amount of L2 for comparison. Serum collected from mice immunized with 4 µg of 11-88x5 had a mean HPV16 L2 17–36 peptide ELISA titer of 2960, which is ≈12-fold higher than the mean serum titer of the GST-HPV16L1Δ-L2x3-immunized group (230) (Figure 2B). As compared with GST-HPV16L1Δ-L2x3 antisera, the antisera to the 11-88x5 fusion was also more cross-reactive with L2 neutralizing epitopes of HPV types not utilized in making the 11-88x5 construct, notably HPV31, HPV35, HPV45, and HPV58.

### *In vivo* PsV vaginal challenge of capsomer-immunized mice

**Figure 3:**
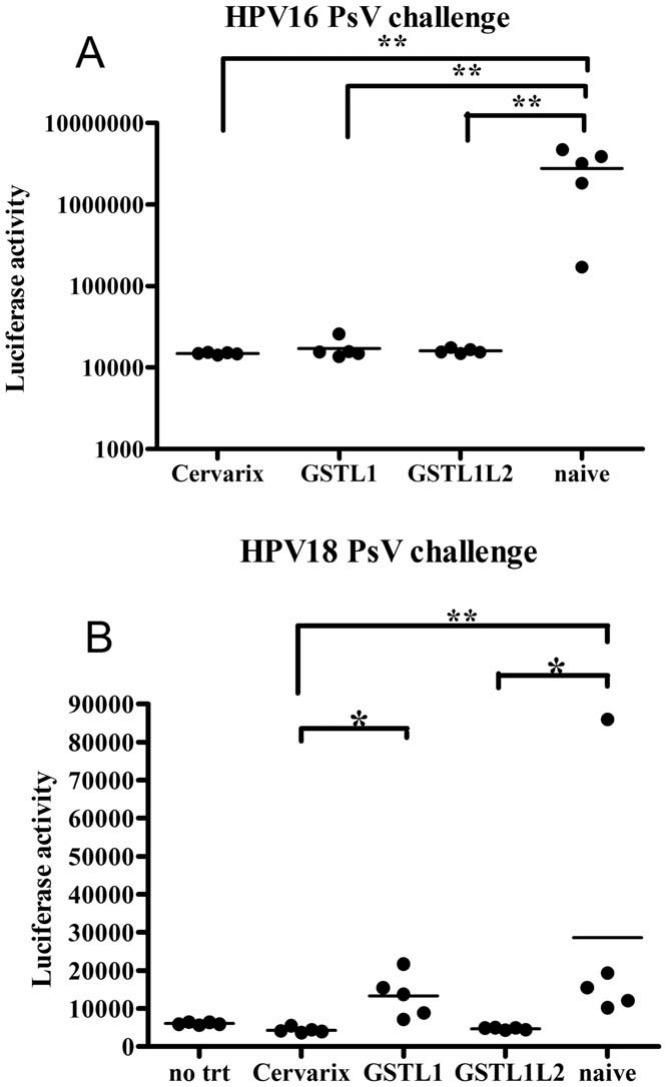
Vaginal challenge of mice with HPV PsV after immunization with capsomers. Mice were subcutaneously immunized with GST-HPV16L1Δ, GST-HPV16L1Δ-L2x3 or Cervarix® three times at two-week intervals. Three and five weeks after the last immunization, the mice were challenged with either HPV16 (A) or HPV18 (B) PsV carrying a luciferase reporter, respectively. Three days after challenge, the mice received an intravaginal instillation of 20 µl of D-luciferin and were imaged with a Xenogen IVIS 100 system (image captured for 10 min post-installation of luciferin). Image analysis in each experiment was based on the average measurement (photons/s/cm^2^) collected from each selected equally-sized area (A, 1.9 cm^2^; B, 0.6 cm^2^) in the vaginal tract.

Because vaccination with both capsomer vaccines elicited robust HPV16 neutralizing antibody titers, the mice were subjected to vaginal challenge with HPV16 pseudovirus carrying a luciferase reporter to facilitate the measurement of infectivity *in vivo*. Three days after intravaginal challenge with HPV16 PsV, the non-immunized animals exhibited a high mean luciferase activity (Figure 3A). Conversely, mice immunized with either GST-HPV16L1Δ or GST-HPV16L1Δ-L2x3 exhibited background levels of luciferase reporter gene activity similar to that of Cervarix®-immunized mice, which is consistent with complete protection. The background luciferase levels in the mice immunized with GST-HPV16L1Δ, GST-HPV16L1Δ-L2x3 or Cervarix® were not significantly different (Figure 3A).

Vaccination with GST-16L1Δ-L2x3 elicited L2-specific antibody responses, but no cross-neutralizing antibody responses were detected for HPV18, HPV31 or HPV45. However, previous studies indicate that the antibody levels necessary for protection are low and that the current neutralization assay may lack sensitivity for L2-specific neutralizing antibodies [37]. In order to determine if vaccination with GST-HPV16L1Δ can induce cross-protection, or if fusion of L2x3 to the C-terminus of GST-HPV16L1Δ can enhance cross-protection, immunized mice were vaginally challenged with HPV18. Despite using a greater challenge dose of HPV18 PsV (8.3 µg of L1) than HPV16 PsV (1 µg of L1), it is noteworthy that HPV16 PsV are dramatically more infectious in mice than HPV18 PsV (Figure 3B). Nevertheless, naïve mice exhibited a clear signal after HPV18 PsV challenge, while mice vaccinated with Cervarix® that contains HPV18 L1 VLPs, exhibited background levels, which is consistent with complete protection (Figure 3B). Vaccination with GST-HPV16L1Δ capsomers failed to provide significant protection against HPV18 challenge, suggesting that capsomers, like L1 VLPs, trigger a type-restricted protective immune response. Conversely, vaccination with GST-16L1Δ-L2x3 provided robust protection against HPV18 challenge, which was not significantly different from that achieved with Cervarix® (Figure 3B).

## Discussion

Vaccination with GST-HPV16L1Δ was as effective as Cervarix® in protecting mice against vaginal challenge with HPV16, although inducing neutralizing antibody titers approximately seven-fold lower than Cervarix®. However a more optimized capsomer formulation may reduce or negate this difference, and the difference with Gardasil® may be less [38]. It should be noted, however, that very low titers of antibodies appear to be sufficient to protect mice from vaginal challenge [37], as illustrated by the cross-protection of mice against HPV18 by vaccination with GST-16L1Δ-L2x3. In this case, the mean titer of HPV18 neutralizing antibodies was below the threshold of detection, yet a similar level of protection was observed in mice vaccinated with Cervarix®, which exhibited very high titers of antibodies. It is possible that the difficulty in detecting L2-specific protective antibodies reflects differences in the exposure of L2 during *in vitro* infection of 293TT cells as compared with *in vivo* infection of the genital mucosa, or dramatically lower levels and/or avidity as compared to L1-specific neutralizing antibodies. The presence of L2-reactive antibodies in the sera of mice immunized with GST-HPV16L1Δ-L2x3 was detectable at low titer by ELISA, suggesting the latter possibility. The antibody responses to the conserved L2 neutralizing epitope comprising residues 17–36 [24] were approximately 12-fold lower in mice immunized with GST-HPV16L1Δ-L2x3 as compared mice immunized with L2 11-88x5. This result suggests that fusion of L2 with L1 does not enhance, and may compromise the response to L2. When L2 is simply mixed with L1 capsomers, no interference is observed [21], whereas L2 is subdominant to L1 when they are co-assembled into VLPs [30]. Thus there are several efforts to substitute the immunodominant L1 epitopes with a cross-neutralizing L2 epitope to render L2 more immunogenic like L1 [39], [40], [41]. Because vaccination with GST-16L1Δ protected against challenge with HPV16 but not HPV18 PsV, the protection provided by L1 capsomers appears to be type-restricted as seen for L1 VLPs, and exposure of the sides of the capsomers by preventing assembly does not uncover cross-protective L1-specific epitopes. Clearly, the alternate approach to achieve broad protection is to generate a highly multivalent L1 capsomer-based vaccine, and capsomers have already been produced for HPV11, HPV16, HPV18 and HPV35 [42]. Nevertheless, the fusion of an L2 multimer to the C-terminus of L1 did not negatively impact the L1 responses, and it provided broader protection.

The cost of manufacture remains a major, although not the only, barrier to global implementation of HPV vaccines [2]. The ability to produce very high yields of capsomers in a low cost bacterial expression system suggests the potential for cost reduction. The N-terminal deletion in L1 enhances production, and fusion to GST facilitates capsomer purification. The C-terminal deletion removes the arm that otherwise coordinates with adjacent capsomers within a VLP as well as the L1 DNA-binding domain, thereby obviating the need for disassembly/reassembly to remove contaminating host DNA during VLP purification [43], [44], [45].

Herein we examined the immunogenicity of L1 capsomers on which the GST was retained. Although a tag such as GST would typically be removed from an antigen being considered for clinical application, the GST was retained because it has potential as an anti-schistosome immunogen [46], [47]. Schistosomiasis is the second major parasitic disease after malaria and is an important public health problem in many non-industrialized countries. Furthermore urinary schistosomasis is a trigger for bladder cancer. The GST used in this study for affinity purification is derived from *S. japonicum*. GST derived from *S. hematobium* is currently in phase III testing (clinicaltrials.gov; identifier NCT00870649) to examine its value as a therapeutic vaccine (Bilhvax) in children exposed to urinary schistosomiasis [47]. Therefore, the inclusion of GST in the capsomer vaccine not only facilitates the purification process, it also confers the potential to trigger immunity at low cost against both papillomavirus and schistosome infections, two carcinogenic infections that cause significant mortality and morbidity in developing countries [12].

## Materials and Methods

### Ethics Statement

This study was carried out in strict accordance with the recommendations in the Guide for the Care and Use of Laboratory Animals of the National Institutes of Health. All animal studies were performed with the prior approval of the Animal Care and Use Committee of Johns Hopkins University (protocol MO08M19).

### Cells, plasmids, media and growth conditions

Plasmid pGEX-HPV16 L1Δ (encoding amino acids 36–502 from GenPept accession number NP_041332.1) was constructed for the expression of the deletion mutant HPV16 L1ΔN9ΔC29 with an N-terminal GST tag. The HPV16 L1 gene was codon optimized for expression in *Escherichia coli* and synthesized by Genscript with a BamHI site at its 5′ end, and two stop codons and an XhoI site at its 3′ end. It was then cloned into the BamHI/XhoI sites of pGEX-4T-2 (GE Healthcare) and verified by sequencing.

Plasmid pGEX-HPV16 L1Δ-L2x3 was constructed by amplifying HPV16 L1ΔN9ΔC29 from the aforementioned plasmid and reinserting the L1 segment into pGEX-4T-2 using the BamHI and EcoRI restriction sites. The L2 segment of this plasmid was created synthetically using overlapping oligonucleotides that were assembled and amplified using PCR. A sequence containing amino acids 12–46 from HPV18 L2, amino acids 13–46 from HPV31 L2 and amino acids 12–46 from HPV45 L2 (GenPept accession numbers AAP20600.1, AAA92893.1 and AAY86493.1, respectively) in tandem were submitted to the online program DNAWorks (http://helixweb.nih.gov/dnaworks/). This sequence also contained five glycines preceding the HPV18 L2 sequence and two glycines in between the HPV18 and 31 and HPV31 and 45 sequences. The DNAWorks output included 16 oligonucleotides optimized for expression in *E. coli*. These oligonucletoides were assembled by PCR in a reaction containing 100 ng of each oligo, 200 mM of each dNTP, 1x *PfuTurbo* reaction buffer and 2.5 U *PfuTurbo* (Stratagene). The assembly reaction was performed as follows: 2 min at 95°C, 25 cycles of 30 sec at 95°C, 30 sec at 55–65°C and 1 min at 72°C followed by 10 min at 72°C. Each of these reactions (1 µl as a template) was then amplified using the outermost oligonucleotides as forward and reverse primers (100 ng each). The amplification reaction was performed as follows: 2 min at 95°C, 25 cycles of 30 sec at 95°C, 30 sec at 62°C and 1 min at 72°C followed by 10 min at 72°C. The assembly reactions performed between 60 and 65°C resulted in the appropriately sized product. This product was digested and ligated into the pGEX-4T-2 vector containing HPV16 L1Δ between the EcoRI and NotI sites. The plasmid was verified by DNA sequencing.

### Purification of capsomers

Truncated HPV16 L1 was expressed as a GST fusion protein either alone or with the tandem HPV L2 epitopes in BL21(DE3) *E. coli* (Stratagene). A single colony of transformed bacteria was inoculated into 50 ml of Terrific Broth (TB) (1.2% Tryptone, 2.4% yeast extract, 0.5% glycerol, 17 mM KH_2_PO_4_ and 72 mM K_2_HPO_4_) containing 100 µg/ml ampicillin and grown overnight at 30°C. The 50 ml overnight culture was then used to inoculate 500 ml of fresh selection medium to an optical density (OD)_595_ of 0.1, and this culture was grown at 37°C until the OD_595_ reached 4.0. The temperature was decreased to 25°C, 200 µl of 0.5 M IPTG was added and the culture was grown to an OD_595_ of 8. The cultures were then split into two bottles of 250 ml each, and the cells were harvested by centrifugation at 5000 rpm for 15 min at 4°C. The cell pellets were frozen at −20°C until use.

The cells were lysed, and the fusion proteins were purified via affinity chromatography. Each cell pellet was resuspended in 100 ml of ice-cold buffer L (50 mM Tris-HCl pH 8.0, 0.2 M NaCl, 1 mM EDTA) with 5 mM DTT, 1 mM PMSF and 1x complete protease inhibitor cocktail (Roche). The cell suspension was homogenized using a French pressure cell (Thermo Scientific) at 2,000 psi to complete cell lysis. DNase I (40 U/ml), 2 mM ATP and 10 mM MgCl_2_ were added to the lysed bacteria, and the lysate was rocked for 1 h at room temperature. Solid urea was then added to a final concentration of 2.3 M, and the lysate was again rocked for 1 h at room temperature. The lysate was dialyzed overnight at 4°C into two changes of buffer L/5 mM DTT without urea, centrifuged at 25,000×g for 20 min at 4°C and passed through a 0.2 µm filter. A volume of 50 ml of clarified lysate was applied to a glutathione sepharose column (GSTrap FF 5 ml column, GE Healthcare) at 0.5 ml/min using an ÄKTA FPLC system (GE Healthcare). The column was washed with four column volumes each of buffer L with 2 mM DTT and buffer L with 2 mM DTT and 0.01% Tween 80. The GST-tagged proteins were eluted with buffer L with 2 mM DTT, 0.01% Tween80 and 10 mM reduced glutathione. SDS-PAGE analysis of the eluted fusion protein demonstrated GST-HPV16L1Δ or GST-HPV16L1Δ-L2x3 and free GST as the major components. The concentration of the GST-16L1Δ and GST-16L1Δ-L2x3 proteins in the samples used for vaccination were estimated by SDS-PAGE in comparison to BSA standards. The conformation of these proteins was verified by ELISA with the H16.V5 antibody [48], which specifically reacts with a conformationally-dependent neutralizing epitope on the L1 surface.

### Generation of HPV pseudoviruses (PsV)

Pseudoviruses were generated as previously described [49]. Briefly, plasmids pshellHPV16L1L2, pVitroHPV18L1L2, pVitroHPV31L1L2 or pVitroHPV45L1L2 were transfected with either a luciferase or alkaline phosphatase (SEAP) reporter gene plasmid into 293TT cells [49] using TransIT-2020 transfection reagent (Mirus). Three days after transfection, cell pellets were collected and rinsed with DMEM and Dulbecco's PBS. The pellets were resuspended in a small volume of DPBS-Mg (DPBS supplemented with 9.5 mM MgCl_2_) and then transferred into siliconized tubes. Cells were pelleted by low speed centrifugation, and the supernatant was discarded. For PsV maturation, an equal volume of lysis buffer (DPBS-Mg supplemented with 0.5% of Brij 58 and 0.2% benzonase) was added to the cell pellet, which was allowed to incubate at 37°C for 24 h. After maturation, lysates were adjusted to 850 mM NaCl and extracted with high salt buffer (DPBS with 0.8 M NaCl). Lysates were clarified by centrifugation at 10,000×g for 10 min, loaded onto an Optiprep step gradient (27, 33 and 39%) and spun at 40,000 rpm in a SW40 rotor for 16 h at 16°C. After centrifugation, 0.5 ml fractions were collected from the top of the gradient. A sample representing each fraction was diluted and tested on 293TT cultures [49] for reporter gene expression. Fractions with highest reporter gene expression were pooled and used for the serum neutralization assay and the *in vivo* challenge experiments.

### Immunization of mice

Inbred 4- to 6-week old female BALB/c mice (NCI) in groups of five or 10 were immunized subcutaneously three times at two-week intervals. The immunogens used were 25 µg of GST-HPV16L1Δ, 25 µg of GST-HPV16L1Δ-L2x3 or the indicated doses of the 11-88x5 HPV L2 fusion peptide. Each dose of immunogen (50 µl) contained comparable adjuvant (50 µg aluminum hydroxide and 5 µg 3-O-desacyl-4′-monophosphoryl lipid A [MPL]) to 1/10^th^ of a human dose of the Cervarix® vaccine.

### Measurement of neutralization antibody titers

Serum samples collected from each mouse two weeks after the final immunization were serially diluted two-fold in culture medium, mixed with an equal volume of HPV pseudovirions containing either luciferase or SEAP reporter genes, and incubated at 37°C for 2 h. These samples were each added to 293TT cell cultures (3×10^4^ cells/well), which were then incubated for 72 h [49]. To measure luciferase expression, culture media were removed from each well and 1x Cell Culture Lysis Reagent (CCRL, Promega) was added to lyse the cell monolayer for 15 min at room temperature on a rocking platform. A volume of 20 µl of the lysate from each well was transferred to a black microtiter plate followed by the addition of 50 µl/well of 1x luciferase substrate (Promega). The plates were scanned with the GloMax®-Multi Detection System (Promega). For SEAP measurement, 40 µl of cell-free supernatant was collected from each well, mixed with 20 µl of 0.05% CHAPS, heated at 65°C for 30 min and then cooled on ice. A volume of 200 µl/well of p-nitrophenyl phosphate (pNPP) substrate (2 M diethanolamine with 1 mg/ml of pNPP) was added to each well for color development at ambient temperature, and absorbance data was collected with an automatic ELISA plate reader (Bio-Rad). Data was collected when the absorbance from the wells incubated with only PsV (ODpsv) had an OD_405_>1. The highest dilution of serum that resulted in 50% or more reduction in ODpsv was defined as the endpoint neutralization titer and expressed as its reciprocal value.

### Determination of ELISA antibody titers

L2 antigens (HPV16 L2 amino acids 11–200 or full length HPV18 L2 or HPV31 L2 were generated in *E. coli* as 6xHis fusions [35], or L2 amino acids 17–38 of HPV16, 17–36 of HPV31, 17–36 of HPV35, 16–35 of HPV45 or 16–35 of HPV58 chemically synthesized as a peptide) were coated onto an ELISA plate (MaxiSorp; Thermo Scientific) at 100 ng of protein or 500 ng of peptide per well and incubated at 4°C overnight. Coated plates were blocked with PBS-BSA (1x PBS supplemented with 1% BSA) at 37°C for 1 h and then incubated with 100 µl of the diluted serum samples (two-fold serial dilution from 1∶50) at ambient temperature for 1 h. Plates were then washed three times with PBST (1x PBS with 0.01% Tween 20), and bound antibody was detected with horseradish peroxide-conjugated sheep anti-mouse IgG antibodies (GE Health) followed by further washes with PBST and development with the 2,2′-azinobis[3-ethylbenzothiazoline-6-sulfonic acid]-diammonium salt (ABTS) substrate (Roche). The cutoff OD value (OD_cutoff_) for the ELISA assay was set at three times the average absorbance detected from five naïve mouse serum samples. The highest dilution of serum sample that had an OD value equal to or above the OD_cutoff_ was defined as the endpoint ELISA titer.

### *In vivo* PsV vaginal challenge

Four days before challenge, female Balb/c mice were injected subcutaneously with 3 mg of medroxyprogesterone (Depo-Provera; Pfizer) to synchronize their estrus cycles [50]. Each challenge dose was comprised of 10 µl PsV mixed with 10 µl of 3% carboxymethyl cellulose (CMC). The dose was delivered twice into vaginal vault, the first 10 µl just prior to and the second 10 µl just after treatment with a cytobrush cell collector. The cytobrush cell collector was inserted into the vaginal vault and turned both counter-clockwise and clockwise 15 times while the mice were anesthetized. Three days after PsV delivery, the mice were again anesthetized and 20 µl of luciferin (7.8 mg/ml) was deposited in the vaginal vault. Luciferase signals were acquired for 10 min with a Xenogen IVIS 100 imager, and analysis was performed with Living Image 2.0 software.

### Statistical analysis

Data were analyzed with one-way ANOVA, or the Kruskal-Wallis test and multiple comparison methods (GraphPad 4).
